# Enhanced accuracy of NIRS-vascular occlusion testing through incorporation of conduit artery diameter

**DOI:** 10.1088/2515-7647/ae4862

**Published:** 2026-02-27

**Authors:** Soongho Park, Julie Mathew, Eric Leifer, Sharon Osgood, Claudia Gomez, Hans Ackerman, Yogendra Kanthi

**Affiliations:** 1 Division of Intramural Research, National Heart, Lung, and Blood Institute, National Institutes of Health, 10 Center Dr, Bethesda, MD 20814, United States of America; 2 Intramural Research Program, National Institute of Allergy and Infectious Diseases, National Institutes of Health, 10 Center Dr, Bethesda, MD 20814, United States of America

**Keywords:** brachial artery, hemodynamic parameters, inter-individual variability, microvascular reactivity, near-infrared spectroscopy, quantitative vascular assessment, vascular occlusion test

## Abstract

Near-infrared spectroscopy with vascular occlusion testing (NIRS-VOT) offers a non-invasive approach for real-time assessment of tissue oxygenation and vascular function. However, its clinical application remains limited, in part due to substantial inter-individual variability in anatomical and physiological characteristics. To identify potential sources of variability, we explored whether incorporating conduit artery size—specifically baseline brachial artery diameter (BrAD)—into the NIRS-VOT data analysis could enhance physiological interpretation and reduce measurement error. We analyzed NIRS-VOT responses from 16 healthy participants recruited to the NIH Clinical Center (NCT06552767 and NCT03538639), incorporating individual BrAD measurements as a covariate. Strong to moderate Spearman’s rank-order correlations were observed between BrAD and dynamic perfusion parameters, including the desaturation rate ($\rho $ = 0.77, *p* = 0.0005) and resaturation rate ($\rho $ = 0.79, *p* = 0.0003). These findings suggest that larger brachial arteries are associated with greater oxygen extraction during occlusion and faster reoxygenation during reperfusion. Across parameters, BrAD explained a substantial proportion of the variance in NIRS-VOT outcomes. When BrAD was included as a covariate, the unexplained variability in the desaturation and resaturation rates was reduced to 28% and 34% of the total variance, respectively, indicating that accounting for conduit artery size substantially decreases residual variability and enhances the interpretability of these dynamic responses. Visual comparison also indicated that incorporating BrAD helped clarify response patterns and reclassify outliers. By reducing inter-individual variability and explaining a greater share of the physiological response, the BrAD-informed analysis enhances the interpretability and consistency of NIRS-VOT measurements. Integrating vascular anatomy into NIRS-VOT analysis may improve the detection of subtle vascular dysfunction and strengthen its diagnostic utility. Future research involving larger and more diverse cohorts, and additional vascular territories are needed to validate and expand these findings.

**Abbreviations**
BrA: Brachial artery
BrAD: Brachial artery diameter
NIRS: Near-infrared spectroscopy
VOT: Vascular occlusion test

## 1 Introduction

NIRS is a non-invasive technique that enables real-time monitoring of tissue oxygenation and hemodynamics by examining the interactions between tissue and light, including absorption, scattering, and transmittance [1]. Owing to its simplicity, portability, and resilience to motion artifacts, NIRS has been applied across diverse fields, ranging from agricultural monitoring [2], meat quality measurement [3], clinical diagnostics [4–8]. Since the 1980s, NIRS has been increasingly used as a non-invasive method to measure vascular function in clinical trials, and has been proposed as a functional biomarker tool for use in vascular diseases [9, 10].

Among its clinical applications, one widely used application is the VOT [11], which employs NIRS to assess microvascular reactivity by monitoring the rate of oxygen resaturation following a brief period of ischemia. The NIRS-VOT method has been used in diverse clinical populations, including assessments of muscle oxygenation during exercise in peripheral artery disease [12], comparisons of vascular reactivity in hyperglycemic states between normal-weight and obese individuals [13], and evaluations of vascular reactivity in hypertension [14].

The simplicity and compactness of the NIRS-VOT protocol make it attractive for bedside or outpatient use, but its adoption in clinical settings has been limited by substantial inter-individual variability. The interpretation of NIRS signals can be complicated by technical factors, including the anatomic location of the sensor, variable sensor components, and structural features of the NIRS device [15], as well as the physical and psychological state of the subject [16, 17]. Anatomic factors are often overlooked but potentially important variables to consider in interpreting tissue perfusion measures. One such parameter for evaluation is the size of the BrA, which is the conduit vessel supplying blood to the tissue monitored by the NIRS sensor. Although the NIRS-VOT technique is intended to probe microvascular responses, the optical signal may be substantially affected by nearby major vessels, leading to inconsistent or confounded measurements [18–20].

To improve the reliability and interpretability of NIRS-VOT, recent studies have attempted to integrate dynamic macrovascular responses with microvascular measurements. Notably, McLay *et al* [19] and Soares *et al* [20] demonstrated moderate correlations between the reperfusion slope in NIRS-VOT which reflects the rate of oxygen delivery restoration following occlusion and the percent change in large artery diameter during flow-mediated dilation (%FMD), a commonly used marker of endothelial function (Pearson *r* = 0.63, *p* = 0.003 at the popliteal artery, Pearson *r*= 0.66, *p* = 0.001 at the BrA, respectively). These findings observed across different anatomical sites raise the possibility that macrovascular properties may influence microvascular hemodynamic recovery. However, NIRS-VOT studies share two important limitations. First, they primarily investigated dynamic vascular responses, rather than considering whether static anatomical features like resting artery diameter could better explain inter-individual differences in NIRS outcomes. Second, and perhaps more critically, they relied on relatively homogeneous populations, such as young, healthy individuals with restricted BMI ranges, recruited under strict protocols-without accounting for natural anatomical variability. Although these recruitment strategies were not specifically designed to control conduit artery size, they may have indirectly limited population sampling and generalizability, as differences in body shape or habitual physical activity can influence vascular structure and thereby affect NIRS outcomes. While this approach reduces variability in controlled research settings, it may also limit broader applicability of NIRS-VOT as a clinical tool. In support of this, van Beekvelt *et al* [21].  showed that adipose tissue thickness can substantially confound NIRS measurements and suggested that future studies take anatomical differences into account. This idea supports our rationale for considering conduit artery diameter as a potential anatomical factor influencing NIRS-VOT outcomes.

In light of these limitations, we propose an alternative approach that considers a simple, static anatomical feature—the resting baseline the BrAD—in relation to NIRS-VOT outcomes, specifically the reperfusion slope. We hypothesize that upstream conduit artery characteristics, such as BrAD, influences downstream microvascular oxygenation dynamics measured by NIRS-VOT. Although NIRS does not provide absolute measurements of blood flow, it captures microvascular oxygenation dynamics shaped, in part, by macrovascular anatomy. In this study, BrAD served as a surrogate maker to account for inter-individual variability, aiming to improve the interpretability of NIRS-based vascular assessments by considering upstream anatomical features.

To evaluate the importance of macrovascular anatomy in NIRS-VOT, we conducted a study involving healthy individuals, without strict demographic inclusion criteria and deliberately including subjects with a wide range of physiological and anatomical characteristics. This strategy was designed to reflect real-world variability and assess how differences in vascular anatomy, particularly BrAD, could affect inter-individual variability in NIRS-VOT outcomes. To further reduce experimental noise, NIRS measurements were obtained from the thenar eminence—a distal site located downstream of the ipsilateral BrA, reflecting microvascular tissue perfused by its terminal branches. This location was selected because it is a physiologically relevant, downstream vascularized tissue to assess the potential influence of upstream conduit artery anatomy, while also offering relatively uniform skin tone and subcutaneous tissue thickness across individuals [22, 23]. By incorporating vascular anatomy into NIRS-VOT analysis, our approach offers a novel method to improve data interpretation and standardization. This may be especially valuable in comparative studies involving both healthy and diseased populations, where correcting for vessel size could enhance diagnostic sensitivity and help move NIRS-VOT closer to practical clinical application.

## 2 Methods

### 2.1 Subjects

Human subjects: The study was approved by the National Institutes of Health Institutional Review Board (#001871-H, #18-H-0108) and registered at clinicaltrials.gov (NCT06552767, NCT03538639). All participants provided electronic or written informed consent prior to participation. The study was conducted in accordance with the principles of the Declaration of Helsinki. All guidelines for good clinical practice and in the Belmont Report (National Commission for the Protection of Human Subjects of Biomedical and Behavioral Research) were followed. Participants were aged 18 years or older and included a diverse range of individuals without strict BMI criteria in order to establish a baseline for general NIRS-VOT results. A total of 16 healthy volunteers were recruited, all adults without known vascular diseases.

At recruitment, participants were screened for current medication use, medical history, and individuals with cardiovascular disease (including hypertension and tachycardia), those using nicotine products, herbal supplements, or prescription medications, particularly vaso-active or cardiovascular medications, were excluded from the study. All participants were non-smokers. Participants were instructed to abstain from caffeine intake and strenuous physical activity for at least 24 h prior to testing before the experimental session. Resting blood pressure was not measured as part of the protocol; however, prior to data acquisition, participants were given sufficient time to rest in the supine position and all measurements were subsequently performed following a standardized 5 min baseline period to minimize acute physiological variability.

### 2.2 Study design

**Figure 1:**
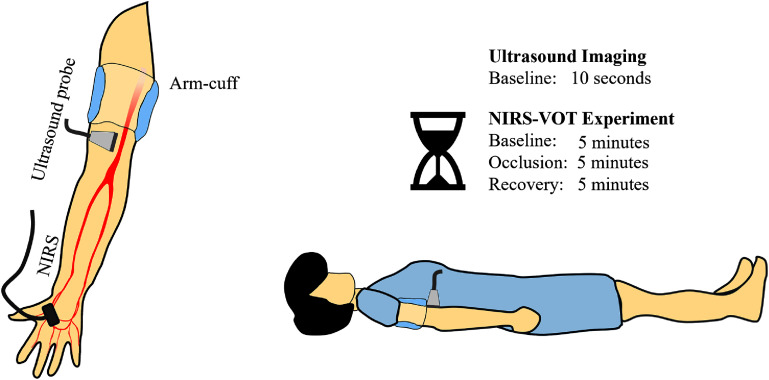
System setup for observing NIRS-VOT signal changes based on the baseline brachial artery diameter (BrAD). A linear ultrasound probe was placed on the upper arm to measure the BrAD, while the NIRS probe was positioned on the thenar.

To investigate the correlation between skeletal muscle oxygenation dynamics and BrAD, NIRS-VOT and ultrasound imaging were conducted simultaneously. Room temperature was maintained at approximately 21 °C using a precision, industrial temperature regulation module, and the participants remained in a comfortable, supine position for the 15 min during of the NIRS-VOT procedure. For the NIRS-VOT procedure, the sphygmomanometer arm air bladder (arm-cuff) was placed on the upper arm to occlude blood flow through the proximal BrA, was inflated to a pressure of 200 mmHg. Inflation and pressure maintenance were controlled using a commercial pressure cuff controller (MOORVMS-PRES, Moor Instruments, UK) under an active monitoring protocol (Post Occlusive Reactive Hyperemia Protocol), ensuring consistent vessel occlusion and minimized pressure loss from air leakage. The experimental system setup is shown in figure 1.

### 2.3 NIRS for tissue oxygenation saturation

The NIRS equipment used was the commercial NIRS device (MOORVMS-NIRS, Moor Instruments, UK). This NIRS device has light sources at two wavelengths: 750 nm and 850 nm, with a maximum light intensity of 12 mW. To allow for independent data analysis, raw data acquired from the device were used for analysis, with a sampling rate of 40 Hz. Before each NIRS-VOT experiment, the calibration status of the device was verified using the NIRS calibration phantom (MOORVMS-NIRS-CAL, Moor Instruments, UK). NIRS-VOT experiments were performed on the upper limb, with measurements taken from the thenar eminence, a commonly used site in peripheral NIRS applications due to its favorable optical properties, clinical accessibility, relatively uniform skin tone [22] and subcutaneous thickness [23],and established relevance for vaso-occlusive testing when required [24]. This location enhances sensitivity and minimizes variability due to superficial tissue characteristics, such as epidermal thickness, melanin concentration (skin tone), and adiposity. To assess deeper tissue oxygenation while reducing the influence of superficial layers, we used a probe with a 30 mm source-detector separation, which was securely affixed to the thenar eminence using double-sided adhesive tape (LAD-NIRS30, Moor Instruments, UK) without obstructing the light path. Although shorter separations (<30 mm) are occasionally used, they are more susceptible to signal contamination from superficial tissue [15, 25].

The probe was passively attached without intentional tissue compression; the probe mass was approximately 19.3 g, corresponding to a contact pressure of less than 1 mmHg when distributed over the probe surface area. This is well below pressures reported to influence NIRS-derived baseline StO_2_ which are typically observed only at substantially higher externally applied pressures (typically $ \unicode{x2A7E} $ 20–30 mmHg) [26]. Therefore, probe pressure was not actively monitored.

To minimize the impact of psychological changes and initialization artifacts from the equipment, sufficient time intervals were included: a 5 min baseline period, a 5 min blood vessel occlusion period, and a 5 min recovery period.

### 2.4 Ultrasound imaging for BrAD

**Figure 2:**
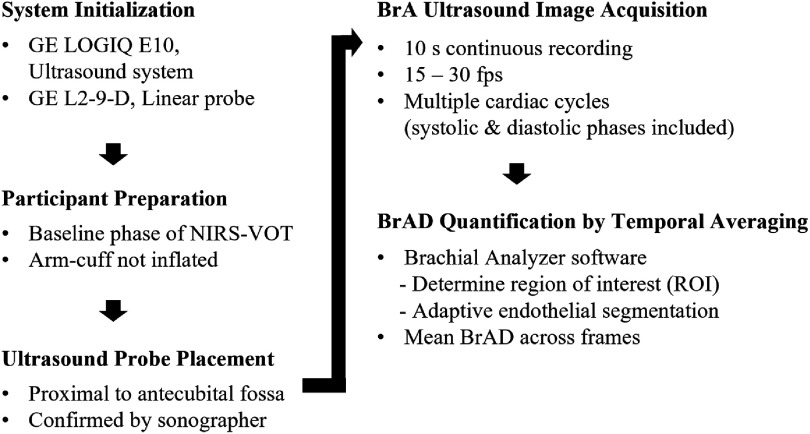
Workflow for brachial artery (BrA) ultrasound image acquisition and brachial artery diameter (BrAD) quantification during the baseline phase of NIRS-VOT.

To investigate the influence of the BrA on the NIRS-VOT outcomes, the BrAD was visualized using a high-resolution multifrequency linear probe (L2-9-D linear array probe, GE HealthCare, USA) with an ultrasound system (LOGIQ E10, GE HealthCare, USA). An overview of the BrAD acquisition and quantification workflow is provided in figure 2. The probe was placed proximal to the antecubital fossa to measure the diameter of the BrA distal to the arm-cuff. The location was determined in real-time by a certified vascular sonographer at the NIH. The antecubital fossa was used as an anatomical reference point across participants, given inter-individual variability in upper-limb length. In practice, the proximal edge of the probe was positioned within approximately 1 cm of the antecubital fossa. The linear probe has an effective imaging footprint of approximately 1 cm $ \times $ 5 cm, and BrAD measurements were derived from the central region of this field of view to ensure consistent sampling of the BrAD segment across participants. The arm-cuff was positioned as proximally as feasible on the upper arm to achieve arterial occlusion while avoiding interference with probe placement. Because the cuff was placed proximal to the imaging site and limb length varied across participants, the distance between the cuff and probe was not explicitly measured. The probe was set to a center frequency of 8 MHz during the observation, referring to the central frequency within the probe’s operating range. BrA images were recorded continuously for exactly 10 s during the baseline phase of NIRS data acquisition, when the arm-cuff was not inflated. ECG-based R-wave gating was not employed; instead, mean BrAD was calculated over both systolic and diastolic phases. The ultrasound system operated at a high temporal resolution, with the frame rate automatically adjusted (approximately 15–30 frames per second) based on imaging depth and contrast optimization. This resulted in acquisition of a large number of frames over the 10 s interval, inherently encompassing multiple cardiac cycles. To ensure consistency across the cohort, the average BrAD over the 10 s period was used for subsequent analysis. Although systolic and diastolic diameters were visually observed during image analysis, measurements were interpreted across cardiac phase to ensure consistency among participants. This approach allowed inclusion of complete cardiac cycles where visible, while maintaining uniform measurement criteria across participants. The acquired video images were analyzed using Brachial Analyzer software [27] (Medical Imaging Applications LLC, USA), which uses an adaptive segmentation algorithm to trace the endothelial border of the BrA and compute the average diameter. The derived BrAD measurements were then used to correlate with the NIRS-VOT outcomes.

### 2.5 NIRS-VOT parameters

**Figure 3:**
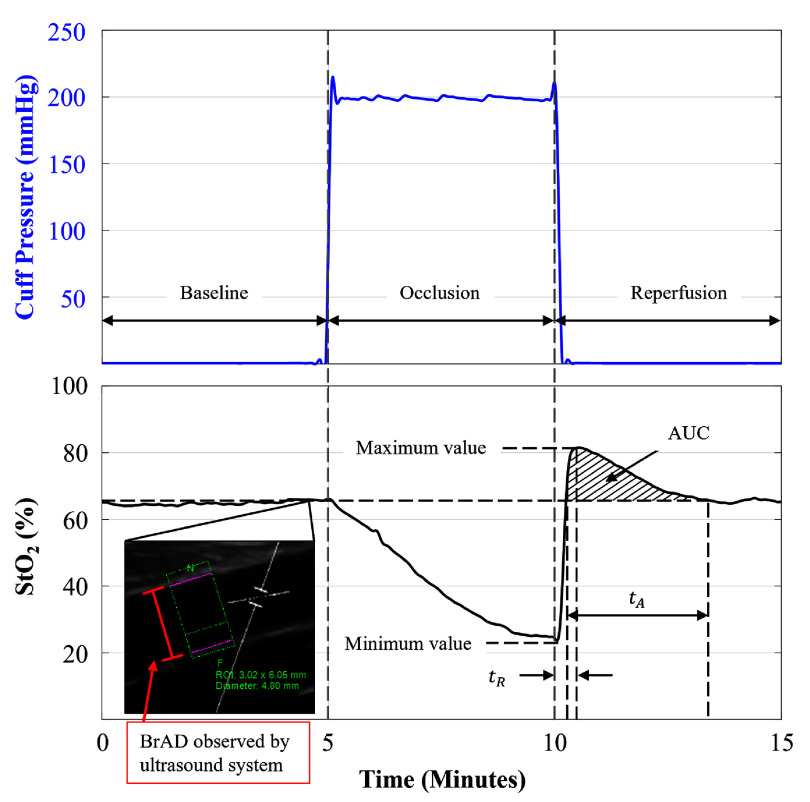
Schematic representation of NIRS-derived tissue oxygen saturation (StO_2_) during a vascular occlusion test. The upper panel shows cuff pressure, and the lower panel shows the corresponding StO_2_ response. The protocol consists of three phases: baseline (5 min resting period prior to ischemia), occlusion (5 min arterial occlusion of the arm), and reperfusion/recovery (5 min period following cuff release). From the StO_2_ traces baseline, minimum, and maximum values were identified. Dynamic reperfusion metrics included resaturation time (*t*_R_), hyperemic recovery time (*t*_A_), and area under the curve (AUC) of the reactive hyperemic response. Inset: representative 10 s acquired brachial artery diameter (BrAD) image showing the region of interest used for analysis.

The key parameters to be observed in the NIRS-VOT are as follows, as shown in figure 3 and described below:

- Baseline (%): The initial oxygen saturation of the tissue (StO_2_) prior to occlusion, averaged over 5 min.
- Minimum (%): The lowest StO_2_ value during the occlusion phase.
- Maximum (%): The highest StO_2_ value after the release of the occlusion.
- Desaturation rate (%/s): The rate at which oxygen is depleted from the tissue during occlusion. A faster rate suggests higher metabolic demand or impaired oxygen delivery,
  \begin{equation*}{R_{{\mathrm{desat}}}} = {\mkern 1mu} \frac{{{\mathrm{Baseline} - \mathrm{Minimum}}}}{{{\mathrm{Occlusion}}{\mkern 1mu} {\mathrm{time}}{\mkern 1mu} \left( {{\mathrm{5}}{\mkern 1mu} {\mathrm{min}}{\mathrm{.}}} \right)}}\end{equation*}

- Resaturation rate (%/s): The rate of tissue oxygenation recovery during reflow from oxygen deprivation during occlusion. A faster rate indicates efficient reperfusion as a surrogate of vascular health.

\begin{equation*}{R_{{\mathrm{resat}}}} = {\mkern 1mu} \frac{{{\mathrm{Maximum} - \mathrm{Minimum}}}}{{{\mathrm{Resaturation}}{\mkern 1mu} {\mathrm{time}}{\mkern 1mu} \left( {{t_{\mathrm{R}}}} \right)}}\end{equation*}

\begin{equation*}{t_R}:{\mathrm{time}}\;{\mathrm{from}}\;{\mathrm{the}}\;{\mkern 1mu} {\mathrm{minimum}}\;{\mathrm{to}}\;{\mathrm{the}}\;{\mathrm{maximum}}\;{\mathrm{St}}{{\mathrm{O}}_{\mathrm{2}}}{\mathrm{value}}\end{equation*}

- Area under the curve (AUC, %$ \cdot $ s): Measures total blood flow recovery following occlusion, reflecting oxygen dynamics, metabolic demand, and vascular function.

\begin{equation*}{\mathrm{AUC}} = \int_{{t_{{\text{A initial}}}}}^{{t_{{\text{A end}}}}} {{\mathrm{(St}}{{\mathrm{O}}_{\mathrm{2}}}-{\text{ Baseline)d}}} t\end{equation*}

- Hyperemic recovery time (*t*_A_, s): The time interval between the upward and downward crossings of the average baseline StO₂ during the reperfusion phase. It represents the duration of the hyperemic response, starting when StO₂ first exceeds baseline and ending when it returns to baseline after reactive hyperemia.

### 2.6 Statistical analysis

Associations between baseline BrAD and variables derived from the NIRS-VOT were evaluated using Spearman’s rank-based correlation coefficient ($\rho $). This non-parametric method was selected as it does not require the assumption that the mean of the residuals is approximately normally distributed, which may not be valid in relatively small samples such as our cohort (*n* = 16).

Although participants represented a range of ethnic backgrounds with varying skin tones, all NIRS measurements were conducted at the thenar eminence, a site considered relatively unaffected by melanin concentration [22, 23]. Therefore, we assumed that potential variation in signal quality due to skin pigmentation was negligible, and thus, data for all racial groups were combined for analysis.

In addition, regression analyses including baseline BrAD were conducted to further characterize relationships between vascular anatomy and NIRS-VOT parameters. Fitted lines with shaded 95% confidence intervals were plotted to visualize these associations, as described in the results section.

All data are reported as mean $ \pm $ standard deviation for normally distributed variables unless otherwise specified. A two-tailed *p*-value $ \unicode{x2A7D} $ 0.05 was considered statistically significant. All statistical analyses were performed using R in RStudio (ver. 2024.12.1 + 563, Post Software, PBC, USA) and MATLAB (ver. R2024a, MathWorks, Inc., USA).

## 3 Results

### 3.1 Subject recruitment

Table 1 summarizes the characteristics of the healthy participants (*n* = 16). The complete dataset, including characteristics of the hypothesis-generating cohort.

**Table 1:** Baseline characteristics of healthy participants.

Variable	Value
Sample size (*n*)	16
Gender	Female: 7, male: 9
Age (*y*)	33 $ \pm $ 9
Race	White (*n* = 9)
	Black (*n* = 4)
	Asian (*n* = 3)
Weight (kg)	74.44 $ \pm $ 24.81
Height (cm)	170.23 $ \pm $ 8.98
BMI (kg m^−2^)	25.33 $ \pm $ 6.00
BrAD (mm)	3.88 $ \pm $ 0.93

### 3.2 BrAD and tissue oxygenation parameters during NIRS-VOT

We first examined integration of NIRS-VOT measurements and baseline BrAD during the initial resting phase of healthy participants. The average BrAD was 3.88 $ \pm $ 0.93 mm. The baseline StO_2_ was 62.54 $ \pm $ 6.11%, with a minimum of 34.14 $ \pm $ 10.24% during occlusion and a maximum of 78.64 $ \pm $ 5.59% during reperfusion. Desaturation and resaturation rates were 0.09 $ \pm $ 0.05%/s and 1.60 $ \pm $ 0.61%/s, respectively. The AUC was 75 940.74 $ \pm $ 47 438.43%∙s. The resaturation time (*t*_R_) was 29.55 $ \pm $ 6.80 s, and reactive hyperemia duration (*t*_A_) was 261.27 $ \pm $ 148.31 s.

### 3.3 Statistical results

**Figure 4:**
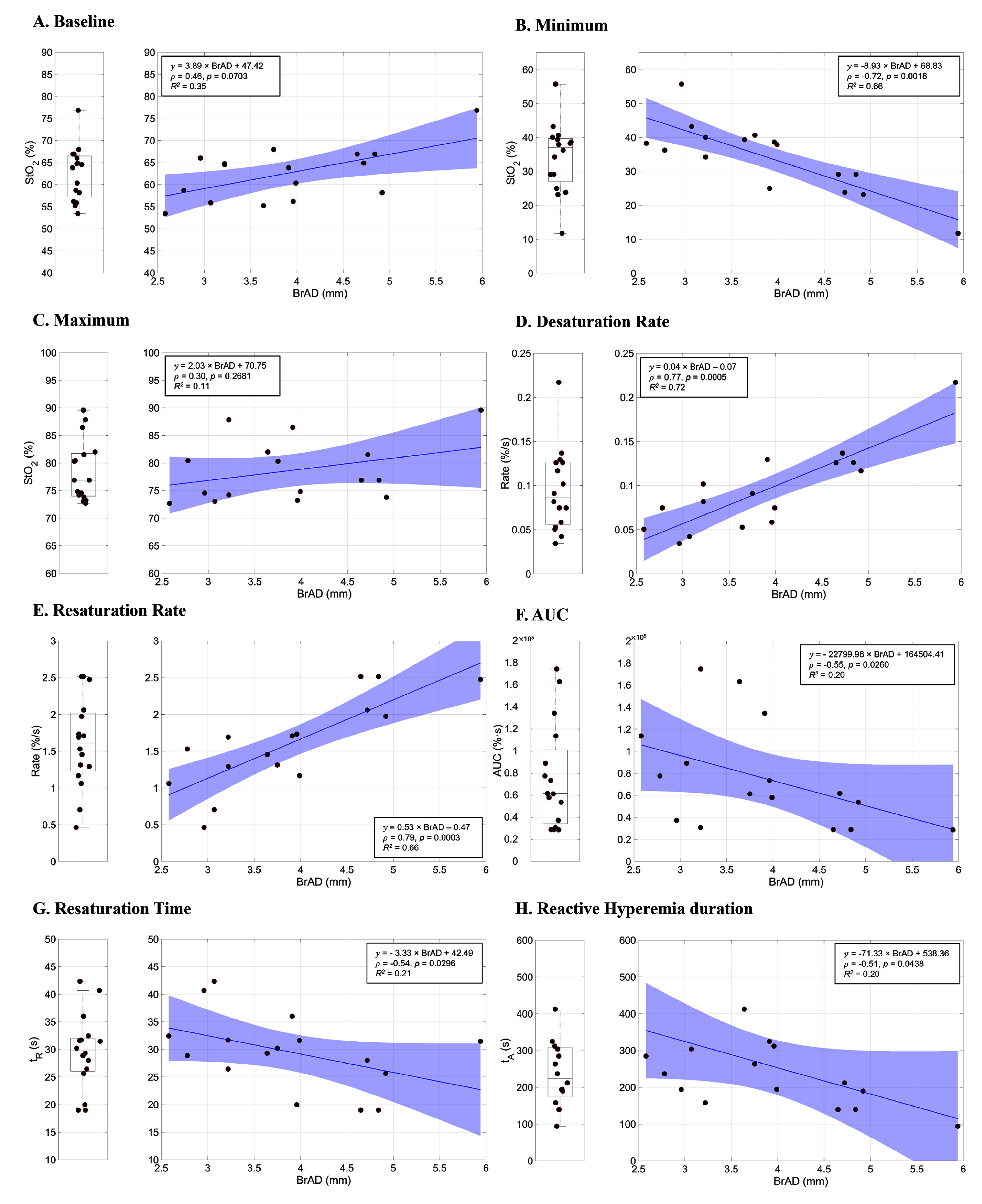
Distribution and BrAD-outcome relationships for NIRS-VOT parameters in healthy participants. For each NIRS-VOT parameter, the left panels show the observed distribution (median and interquartile range), and the right panels illustrate the relationship between the parameter and baseline brachial artery diameter (BrAD) using linear regression with fitted lines and shaded 95% confidence intervals. Panels A-E show StO₂-related measures: (A) Baseline StO₂, (B) minimum StO₂, (C) maximum StO₂, (D) desaturation rate, and (E) resaturation rate. Additional VOT-derived metrics are shown in (F) area under the curve (AUC), (G) resaturation time, and (H) reactive hyperemia duration. These paired views highlight both the distributional variability of each parameter and its association with BrAD.

Each panel in figure 4 presents two complementary analytical perspectives. The left panels show the observed distribution of each NIRS-VOT parameter using boxplots (median and interquartile range, IQRs; 25th–75th percentiles, middle 50%) which summarize variability without accounting for anatomical differences. In contrast, the right panels illustrate the relationship between each parameter and baseline BrAD through scatterplots with fitted regression line and 95% confidence intervals. These representations are intended to visualize distributional patterns (left) versus BrAD-related associations (right), rather than to serve as direct numerical comparisons.

As summarized in table 2, incorporating BrAD into the regression model reduced unexplained variability across nearly all NIRS-VOT parameters. In particular, the unexplained variability in the desaturation and resaturation rates was reduced to 28% and 34% of the total variance, respectively, consistent with the strong positive associations shown in figures 4(D), (E) and physiologically expected patterns of faster oxygen desaturation and reoxygenation in individuals with larger conduit arteries.

**Table 2:** Linear regression analysis of NIRS-VOT parameters in relation to baseline brachial artery diameter (BrAD).

NIRS-VOT parameters	Linear regression model (BrAD as predictor)	Fraction of variance explained (*R*^2^)	*p*-value	Slope SE	Slope 95% CI [lower, upper]
Baseline StO_2_ (%)	3.89 × BrAD + 47.42	0.35	0.0159	1.42	[0.85, 6.94]
Minimum StO_2_ (%)	−8.93 × BrAD + 68.83	0.66	0.0001	1.73	[−12.6, −5.22]
Maximum StO_2_ (%)	2.03 × BrAD + 70.75	0.11	0.2010	1.51	[−1.22, 5.28]
Desaturation rate (%/s)	0.04 × BrAD−0.07	0.72	3.35 × 10^−05^	0.01	[0.0274, 0.0581]
Resaturation rate (%/s)	0.53 × BrAD−0.47	0.66	0.0001	0.10	[0.312, 0.755]
AUC (% $ \cdot $ s, 10^3^)	−22.8 × BrAD + 165	0.20	0.0834	12.2	[−49.0, 3.43]
Resaturation time, *t*_R_ (s)	−3.33 × BrAD + 42.49	0.21	0.0769	1.74	[−7.07, 0.41]
Reactive hyperemia duration, *t*_A_ (s)	−71.33 × BrAD + 538.36	0.20	0.0832	38.2	[−153, 10.7]

SE, standard error; CI, confidence interval.

In figure 4(A) (right panel), baseline BrAD showed a moderate positive association with baseline StO₂ ($\rho $ = 0.46, *p* = 0.0703), suggesting a trend toward higher resting oxygenation among individuals with larger arteries, although this relationship did not reach statistical significance. Minimum StO_2_ showed a strong inverse correlation (figure 4(B); $\rho \,$ = −0.72, *p* = 0.0018), indicating greater deoxygenation during occlusion in participants with larger arteries. Maximum StO_2_ during reperfusion exhibited a weak, non-significant positive trend (figure 4(C); $\rho $ = 0.30, *p* = 0.2681). Both the desaturation rate (figure 4(D); $\rho $= 0.77, *p* = 0.0005) and resaturation rate (figure 4(E); $\rho $ = 0.79, *p* = 0.0003) exhibited strong positive correlations with BrAD, indicating that participants with larger BrAD experienced both faster oxygen desaturation during occlusion and more rapid reoxygenation during reperfusion. Importantly, these patterns were not apparent from the distributional boxplots alone, in which values appeared widely dispersed without evident structure.

Finally, AUC (figure 4(F); $\rho $ = −0.55, *p* = 0.0260), resaturation time (t_R_) (figure 4(G); $\rho $ = −0.54, *p* = 0.0296), and reactive hyperemia duration (*t*_A_) (figure 4(H); $\rho $ = −0.51, *p* = 0.0438) all showed moderate correlation with BrAD, suggesting that individuals with larger conduit arteries recover more rapidly following ischemia.

## 4 Discussion

NIRS-VOT tools have attracted interest in the medical field as a non-invasive, easily deployed system with the capacity to provide real-time data about tissue oxygenation. Despite these advantages, NIRS-VOT has not translated well into clinical diagnostics, largely due to high inter-individual variability in tissue oxygenation response [10, 15, 21, 25]. Variability driven by anatomic and subsequent physiologic differences reduces reproducibility and complicates interpretation, restricting NIRS-VOT primarily to research applications.

To address this limitation, we examined whether baseline BrAD, representing macrovascular capacity for blood delivery, could explain part of the variability in NIRS-VOT outcomes. By incorporating BrAD into the analysis, we observed clear physiological patterns that were not apparent when considering NIRS-VOT parameters alone.

As summarized in figure 4 and shown in table 2, the coefficient of determination (*R*^2^) values indicate that incorporating BrAD explains a substantial proportion of variability in several NIRS-VOT parameters. For example, BrAD accounted for 72% of the variance in desaturation rate and 66% in resaturation rate, meaning that the unexplained variability in these parameters was reduced to 28% and 34% of the total, respectively. These results demonstrate that accounting for conduit-artery size markedly decreases residual variability and clarifies inter-individual differences in dynamic oxygenation responses. These findings support the premise that macrovascular anatomy contributes significantly to downstream microvascular behavior, with larger arteries associated with faster oxygen desaturation during occlusion and more rapid reoxygenation during reperfusion.

The results of both correlation and regression analyses reinforce the physiological relevance of these statistical findings. As shown in table 2 and figure 4, BrAD showed moderate to strong relationships with multiple NIRS-VOT parameters. Beyond the statistical association, these findings reveal that participants with larger arteries are associated with both greater oxygen extraction during occlusion, and faster reoxygenation during reperfusion. Physiologically, this may reflect more efficient perfusion dynamics: individuals with larger arteries may have higher baseline flow capacity, which could support a larger volume of blood in the microvasculature prior to occlusion and enable a more robust hyperemic response upon cuff release. This is consistent with previous findings showing that post-occlusion blood flow and vascular dilation are influenced by initial artery size and compliance [28, 29]. This results in steeper desaturation slopes and more rapid resaturation, both hallmarks of dynamic vascular responsiveness. Furthermore, accounting for BrAD clarified several responses that initially appeared as outliers, indicating that apparent anomalies in unadjusted data may be explained, in part, by underlying anatomic variability.

From a methodological perspective, these findings suggest that incorporating BrAD as an explanatory variable can help account for inter-individual variability in NIRS-VOT outcomes. Future studies could also explore normalizing NIRS-VOT parameters to vessel diameter as an alternative approach for reducing variability.

It is important to consider that certain NIRS-VOT parameters, such as maximum and minimum StO₂ may be susceptible to measurement bias depending on the tissue site or skin characteristics, including skin pigmentation across racial groups. In this study, we chose the thenar eminence because of its relatively uniform skin tone across races [22, 23]. However, other anatomical sites used in many studies, particularly those with greater melanin content (e.g. forearm, upper arm, thigh) may affect absolute StO₂ values. In contrast, desaturation and resaturation rates, which are calculated based on the rate of change between minimum and maximum StO₂ rather than their absolute values, are less likely to be influenced by such bias. This further supports the utility of dynamic, slope-based parameters as more robust and generalizable indicators of vascular function across diverse populations.

In summary, incorporating BrAD into NIRS-VOT analysis reduced unexplained variability and uncovered physiologically meaningful relationships between macrovascular anatomy and microvascular oxygenation dynamics.

## 5 Limitations and future work

The present study was conducted in a relatively small cohort of healthy volunteers and was designed to establish a physiological and methodological framework for interpreting NIRS-VOT measurements by accounting for upstream macrovascular anatomy. This controlled approach enabled a focused evaluation of baseline BrAD as a contributor to inter-individual variability, rather than assessment of diagnostic or prognostic performance. Although statistically significant relationships were observed, confidence intervals around effect estimates may be wide, and the results should therefore be interpreted with appropriate caution.

In addition, although ultrasound probe placement was standardized using anatomical landmarks, with the antecubital fossa serving as the primary reference point, inter-individual in limb anatomy may contribute to residual variability in the BrAD segment imaged across participants.

An important next step will be extension of this framework to larger and more heterogeneous populations, including individuals with peripheral artery disease, diabetes, or obesity, and other conditions characterized by endothelial dysfunction. Such studies will be essential to assess the generalizability of the BrAD–NIRS-VOT relationships identified here and to delineate how anatomical contributors interact with disease-related vascular alterations. While the present findings demonstrate substantial variance explanation for selected NIRS-VOT parameters in healthy individuals, confirmation of these relationships in broader cohorts will further strengthen and refine this framework.

Overall, this work provides a foundation for reducing unexplained inter-individual variability in NIRS-VOT outcomes by incorporating physiologically meaningful anatomical factors, and it offers analytical tools that can be readily adopted and expanded in future physiological, translational, and clinical investigations.

## 6 Conclusion

This study highlights the importance of incorporating baseline BrAD into the analysis of NIRS-VOT data to improve the physiological interpretation of tissue oxygenation responses. By accounting for individual variations in vascular anatomy, we uncovered robust associations between BrAD and several dynamic parameters including minimum StO_2_, desaturation rate, and resaturation rate that were not evident using analysis approaches that did not account for BrAD. These BrAD-sensitive indices provide a more refined lens through which to evaluate vascular function, allowing for clearer differentiation between normal physiological variation and potential dysfunction. Importantly, this anatomically informed approach also allowed for the reinterpretation of data previously regarded as outliers and revealed early indications of impaired vascular responsiveness in older individuals. Although some parameters, such as *t*_R_ and *t*_A_, demonstrated moderate associations with BrAD, the overall findings emphasize the value of integrating structural context into functional assessments. Incorporating vascular anatomy into NIRS-based vascular evaluations may help overcome a key barrier to clinical translation by reducing interindividual variability and enhancing diagnostic precision. Future studies should apply this framework across broader populations and vascular beds to further establish its clinical relevance and generalizability.

This combined statistical and physiological framework enhances the interpretability and reliability of NIRS-VOT measurements, provides a basis for improved metrics in clinical trial design, and may facilitate broader clinical adoption of this technology for evaluating vascular health.

## Data Availability

The data supporting the findings of this study are available in Figshare at https://doi.org/10.25444/nhlbi.31297945. The dataset has been deposited and will be made publicly available upon acceptance of the manuscript. Additional details can be requested by contacting Soongho Park (soongho.park@nih.gov) and Yogendra Kanthi (yogen.kanthi@nih.gov).
